# Compost Amendments Based on Vinegar Residue Promote Tomato Growth and Suppress Bacterial Wilt Caused by *Ralstonia Solanacearum*

**DOI:** 10.3390/pathogens9030227

**Published:** 2020-03-19

**Authors:** Mingming He, Mohammad Shah Jahan, Yu Wang, Jin Sun, Sheng Shu, Shirong Guo

**Affiliations:** 1 Key Laboratory of Southern Vegetable Crop Genetic Improvement in Ministry of Agriculture, College of Horticulture, Nanjing Agricultural University, Nanjing 210095, China; 2017204043@njau.edu.cn (M.H.); shahjahansau@gmail.com (M.S.J.); ywang@njau.edu.cn (Y.W.); jinsun@njau.edu.cn (J.S.); shusheng@njau.edu.cn (S.S.); 2 Department of Horticulture, Faculty of Agriculture, Sher-e-Bangla Agricultural University, Dhaka 1207, Bangladesh; 3 Suqian Academy of Protected Horticulture, Nanjing Agricultural University, Suqian 223800, China

**Keywords:** vinegar residue substrate, tomato bacterial wilt, microbial populations, microbial activities, defense-related enzyme, stress-related gene expression

## Abstract

Tomato bacterial wilt caused by *Ralstonia solanacearum* (RS) is one of the most devastating soil-borne diseases, and compost is to be considered as a resource-saving and environment-friendly measure to control the disease. Herein, a pot experiment was implemented to explore the effects of vinegar residue matrix amendments on the growth performances of tomato seedlings and to examine the suppression ability against bacterial wilt under vinegar residue substrate (VRS), and peat substrate (Peat) with RS inoculation. The results revealed that VRS effectively suppressed the disease incidence of bacterial wilt, increased the number of bacteria and actinomycetes, decreased fungi populations, promoted soil microbial populations and microbial activities, enhanced the growths of tomato seedlings, and modulated defense mechanism. In addition, VRS efficiently inhibited the oxidative damage in RS inoculated leaves via the regulation of excess reactive oxide species (O_2_^•−^ and H_2_O_2_) production, lessening of malondialdehyde (MDA) content, and causing less membrane injury; resulting in enhancements of antioxidants enzymes activities accompanying with modulating their encoding gene expression. The transcription levels of *NPR1*, *PIN2, PR1b, ACO1*, *EDS1*, *PR1B*, *MAPK3*, *PIN2*, and *RRS1* were also modulated with the pathogens inoculated in tomato leaves both in VRS and Peat treatments, which indicated that systemic-acquired resistance possesses cross-talk between salicylic acid, jasmonic acid, and the ethylene-dependent signaling pathway. Besides, the RS inoculation significantly inhibited the growth of tomato seedlings, and all growth indices of plants grown in VRS were considerably higher than those produced in Peat. Taken together, VRS represents a new strategy to control tomato bacterial wilt through boosting the soil microbial populations and microbial activities. Furthermore, VRS promotes the plant immune response to provide a better growth environment for plants surviving in disease conditions.

## 1. Introduction

Tomato (*Solanum lycopersicum* L.) is an important and generally cultivated vegetable crop all over the world [1]. Bacterial wilt is the most destructive vascular phyto-pathological disease in tomato, resulting in severe quality deterioration and economic losses [2]. The disease reduced the fresh fruit yield of hybrid tomatoes by about 26% and total yield losses around 90.62% under severe disease incidence [3]. Currently, the management of bacterial wilt is primarily dependent on crop rotation, grafting, growing disease-resistant cultivars, and the use of bactericides. Nevertheless, these tactics have their shortcomings due to the high survivability of pathogens in complex environmental conditions and instability in disease-resistance breeding [4]. Therefore, it is urgent to find an effective, economical, and environment-friendly method to reduce occurrence of this disease in crops. Compost can arise as a promising alternative to suppress the incidence of bacterial wilt. Adding compost to the soil or substrates is beneficial as it promotes plant development, improves soil quality and structure, and inhibits some soil-borne pathogens [5]. Recently, it has been reported that certain composts used as soil amendments or pot media can protect plants from diseases caused by soil-borne pathogens, such as *Rhizoctonia solani* [6], *Fusarium* spp. [7], *Verticillium dahliae* [8], and *Phytophthora* [9]. Compost mainly utilizes the effects of various microorganisms to mineralize, humus and harmless plant organic residues, so that various complex organic nutrients can be converted into soluble nutrients and humus. The resulting high temperature (60–70 °C) kills the germs, eggs, and weed seeds brought by the raw materials to achieve the purpose of harmlessness. The use of compost is beneficial to plant growth and development, improves soil quality and structure, and inhibits various diseases caused by soil-borne pathogens [10]. Numerous studies have demonstrated that composts are acceptable and popular practices to suppress the soil-borne disease [11], whereas composts from different materials show different inhibitory effects on bacterial wilt [12]. Gorissen et al. [13] recommended that the reduction of *R. solanacearum* population resulted from an alteration of microbial community structure and an increase of bacteria-induced by pig manure. Du et al. [14] have testified that VRS could improve the soil physicochemical characteristics and related enzyme functions to inhibit the survival of *Fusarium oxysporum*. In addition, VRS could augmentation of the microbial activity and microbial diversity to prevent the pathogens attack [15]. Moreover, nutrients and niches become more essential when there is competition take places between pathogens and other microorganisms [16]. Strikingly, VRS can provide enough nutrients and better growth conditions for antagonists to increase their ability to control pathogens [14,17]. Importantly, VRS is a new horticultural organic substrate to meet the ecofriendly agricultural crop production. The inhibiting ability of soil against pathogens rely on multitudinous components namely soil pH, electrical conductivity (EC), organic matter content, mineral nutrition, enzyme activity, microbial diversity and so on, which may cause competition between pathogens and antagonists, and further influence microbial resistance towards pathogens.

Although there is very little evidence found regarding disease controlling ability by VRS, till now, the fundamental regulatory functions of VRS towards bacterial wilt in tomato largely remain unexplored. In the current research, we tried to explore the better understanding suppressive mechanisms of how VRS functions properly against bacterial wilt in the case of disease severity, microbial community structure, soil enzyme activities, plant induced resistance response after RS infection. We found that the application of VRS could promote plant growth, transform soil microbial populations and microbial enzyme activities, enhance defense-related enzyme activities and induce stress-related genes expression, which resulted in increased tolerance to RS in tomato.

## 2. Results

### 2.1. Compost Amendments Delay the Disease Symptoms of Tomato Bacterial Wilt

**Figure 1:**
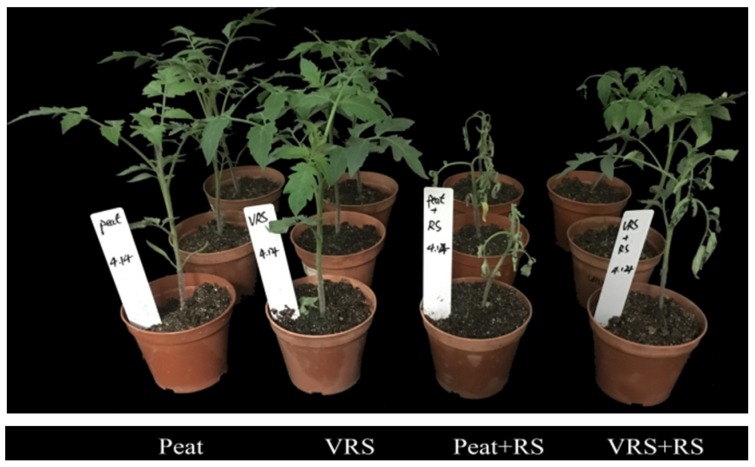
The phenotype of tomato seedlings by inoculation of *R. solanacearum* after 10 days. Peat = peat and vermiculite in a 2:1 ratio (v/v), VRS = vinegar residue substrate, vinegar residue/peat/vermiculite = 3:2:1 (v/v/v). Peat + RS (*Ralstonia solanacearum*) = peat inoculated with pathogen, VRS + RS = VRS poured with pathogen suspensions. Three independent biological replicates were carried out with similar results.

**Figure 2:**
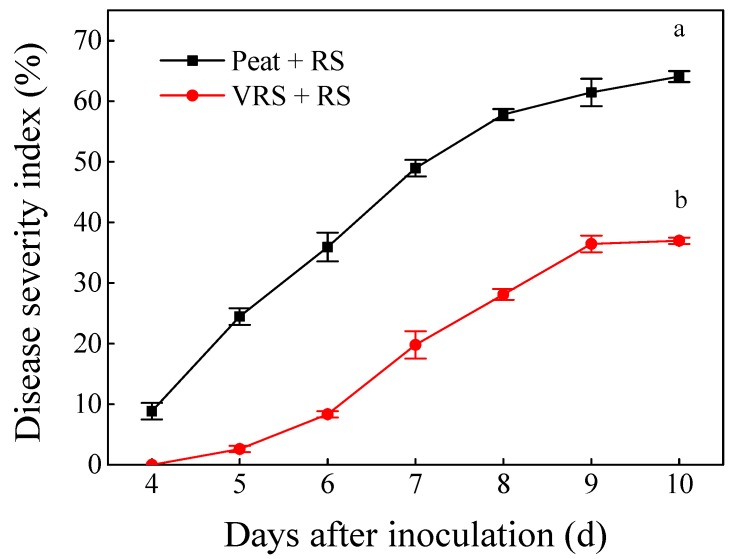
Disease severity index of tomato seedlings in different growth media. Peat = peat and vermiculite in a 2:1 ratio (v/v), VRS = vinegar residue substrate, vinegar residue/peat/vermiculite = 3:2:1 (v/v/v). Peat + RS (*Ralstonia solanacearum*) = peat inoculated with pathogen, VRS + RS = VRS poured with pathogen suspensions. Different letters indicate the significant differences among the treatments at the level of *p* < 0.05, according to Duncan’s multiple range tests. Three independent biological replicates were carried out with similar results.

The disease’s symptoms of tomato seedlings were recorded daily after 10 days of infection with RS. The phenotypic traits showed that disease symptoms conspicuously appeared in tomato leaves when the seedlings were treated with RS in both substrates. The common symptoms caused by RS were higher leaf wilting and dark brown discolorations of the stem (Figure 1). The tendency of the disease index with two treatments (Peat + RS and VRS + RS) displayed differently (Figure 2). Symptoms of wilting began in the Peat + RS-treated seedlings on the fourth day after inoculation, and the disease severity index (DSI) was near to 8.9%. Subsequently, the incidence of disease increased rapidly, and DSI reached 64.1% on the last day of the observation. In contrast, the wilting appearance of VRS+RS-treated seedlings appeared one day later than that in Peat + RS, accompanying 2.6% of DIS, following a slow growth curve, and the final DIS was approximately 40.0%. In brief, VRS decreased the growth of disease index, which reached 42.3% after 10 days compared to the control.

### 2.2. Growth Indices of Tomato Seedling Exposed to RS Inoculation 

As displayed in Table 1 and Table 2, the VRS substrate could facilitate the growth indicators plant height, shoot fresh and dry mass, root fresh and dry matter, stem diameter, leaf area, root surface area, total root length, root volume, and tips of the number of tomato seedlings. However, with the RS inoculation, all growth parameters except for the root mean diameter of tomato seedlings were dramatically decreased both in Peat + RS and VRS + RS-treated seedlings. Meanwhile, the decline degree of Peat + RS was higher than that of VRS + RS. In addition to growth indices, the root vigor also showed the same trend—challenging with RS significantly reduced root vigor in comparison to control, and seedlings planted in VRS + RS increased the root vigor by 56.2% in comparison with the plants grown in Peat + RS (Table 2). 

### 2.3. Enumeration of Culturable Microbial Community and RS Populations

**Figure 3:**
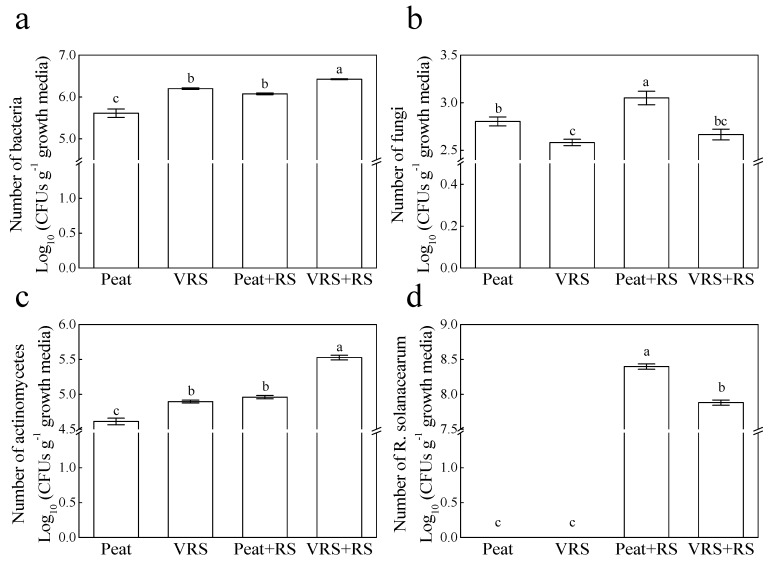
Effects of different growth media on the number of (**a**) bacteria, (**b**) fungi, (**c**) actinomycetes and (**d**) solanacearum in tomato rhizosphere at the end of the experiment. Peat = peat and vermiculite in a 2:1 ratio (v/v), VRS = vinegar residue substrate, vinegar residue/peat/vermiculite = 3:2:1 (v/v/v). Peat + RS (*Ralstonia solanacearum*) = peat inoculated with pathogen, VRS + RS = VRS poured with pathogen suspensions. Different letters indicate the significant differences among the treatments at the level of *p* < 0.05, according to Duncan’s multiple range tests. Three independent biological replicates were carried out with similar results.

At the end of the experiment, we examined the culturable bacteria, fungi, and actinomycetes population to find out the status of microbial community structure. As shown in Figure 3, we observed that the number of bacteria, fungi, and actinomycetes was significantly different (*p* < 0.05) in Peat and VRS media. Increments of three microorganisms were presented in both substrates upon RS inoculation in comparison to no inoculated RS. Meanwhile, bacteria and actinomycetes populations in VRS + RS were remarkably higher, and fungi populations were significantly lower than those in Peat + RS. Moreover, VRS + RS showed remarkably lower RS populations than that in Peat + RS.

### 2.4. Microbial Activity of Substrates after RS Inoculation 

To measure the microbial activities in different substrates, soil fertility, and predict the index of soil disease inhibition ability, we determined the enzyme activities of invertase, urease, catalase, β-glucosidase, proteinase, phosphatase, and FDA hydrolysis. Interestingly, we found that all these enzymes exhibited the same characteristics. The activities of these enzymes in VRS, including challenging with RS, increased to different degrees. Notably, the VRS prominently improved the activities of invertase, urease, proteinase, and β-glucosidase by 257%, 114%, 325%, and 117%, respectively, in respect to Peat + RS (Table 3). What is more, VRS + RS distinctly provoked the activities of urease and proteinase 11.5- and 8.69- fold compared with Peat + RS (Table 3). 

### 2.5. ROS Accumulation of Tomato Seedling after RS Inoculation

**Figure 4:**
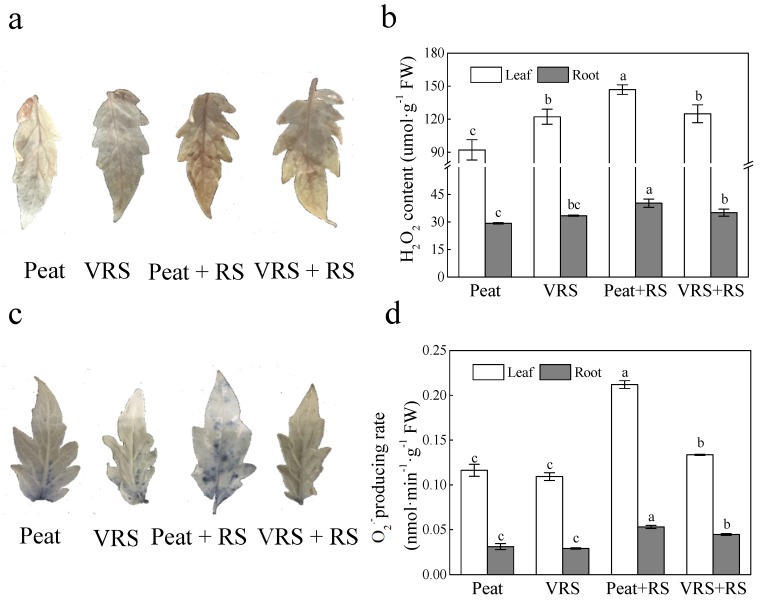
Effects of different compost conditions on (**a**) accumulation of H_2_O_2_ and (**b**) superoxide anion in tomato leaves and (**c**) production rate of H_2_O_2_ and (**d**) superoxide anion in tomato leaves and roots after pathogenic bacteria treatments. Peat = peat and vermiculite in a 2:1 ratio (v/v), VRS = vinegar residue substrate, vinegar residue/peat/vermiculite = 3:2:1 (v/v/v). Peat + RS (*Ralstonia solanacearum*) = peat inoculated with pathogen, VRS + RS = VRS poured with pathogen suspensions. Different letters indicate the significant differences among the treatments at the level of *p* < 0.05, according to Duncan’s multiple range tests. Three independent biological replicates were carried out with similar results.

The leaf ROS accumulation in terms of H_2_O_2,_ and O_2_^·-^ production were increased in plants subjected to RS stress, especially in Peat + RS treatment, than control plants (Figure 4). Deeper tissue staining was observed in tomato leaves in Peat + RS (Figure 3a,c), indicating that the leaves accumulated more ROS. Nevertheless, the differences in staining among other treatments were not noticeable. The H_2_O_2_ contents and O_2_^·-^ generations rate in Peat + RS-treated tomato leaves and roots were substantially more than the control plants (Figure 4b,d). Conversely, the contents of H_2_O_2_ and rate of O_2_^·-^ production in VRS + RS-treated seedlings were significantly lower than Peat + RS-treated seedlings.

### 2.6. Electrolyte Leakage and MDA Content of Tomato Seedlings Exposed to RS Inoculation

**Figure 5:**
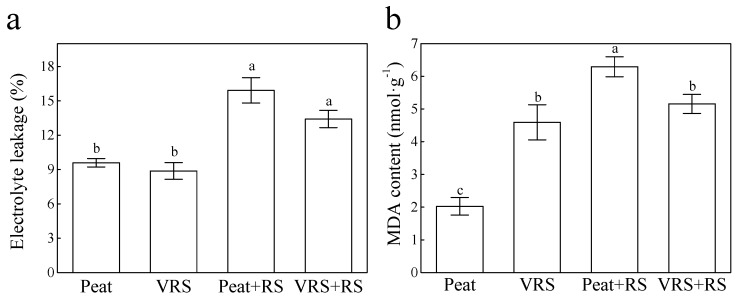
Changes in Change in (**a**) electrolyte leakage (EL) and (**b**) malondialdehyde (MDA) under different compost treatment conditions (Peat, VRS, Peat + RS, VRS + RS) after 10 days of pathogenic bacteria root-irrigations. Peat = peat and vermiculite in a 2:1 ratio (v/v), VRS = vinegar residue substrate, vinegar residue/peat/vermiculite = 3:2:1 (v/v/v). Peat + RS (*Ralstonia solanacearum*) = peat inoculated with pathogen, VRS + RS = VRS poured with pathogen suspensions. Different letters indicate the significant differences among the treatments at the level of *p* < 0.05, according to Duncan’s multiple range tests. Three independent biological replicates were carried out with similar results.

As presented in Figure 5, both electrolyte leakages (ELs), and the content of MDA in tomato leaves of RS inoculation were relatively elevated relative to the control. After injection with RS, VRS + RS-treated seedlings showed less damage due to reducing EL and MDA, whose content decreased by 15.7% and 17.9%, respectively, but their values were more than the control plants (Figure 5).

### 2.7. Studies of Tomato Defense Enzymes after RS Inoculation

**Figure 6:**
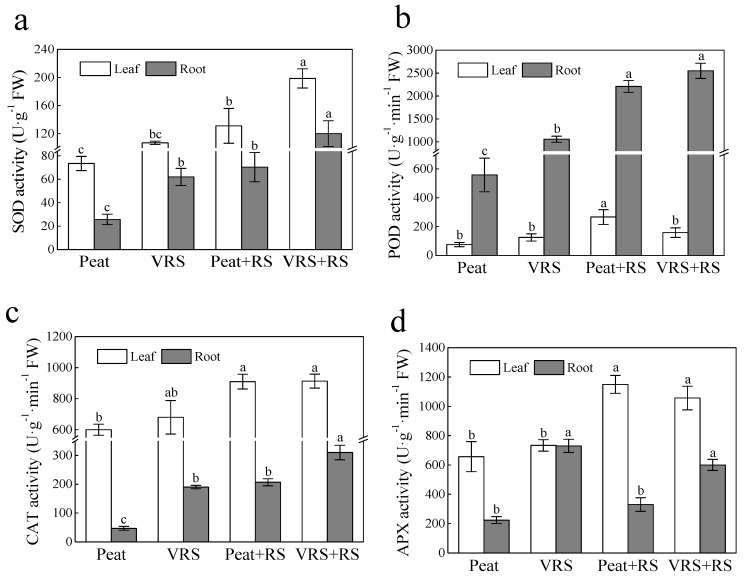
Effects of different growth media on antioxidant enzymes (**a**) Superoxide dismutase (SOD), (**b**) peroxidase (POD), (**c**) catalase (CAT), and (**d**) ascorbate peroxidase (APX) activities in leaves and roots of tomato seedlings after pathogenic bacteria treatments. Peat = peat and vermiculite in a 2:1 ratio (v/v), VRS = vinegar residue substrate, vinegar residue/peat/vermiculite = 3:2:1 (v/v/v). Peat + RS (*Ralstonia solanacearum*) = peat inoculated with pathogen, VRS + RS = VRS poured with pathogen suspensions. Different letters indicate the significant differences among the treatments at the level of *p* < 0.05, according to Duncan’s multiple range tests. Three independent biological replicates were carried out with similar results.

Plants have evolved their inherent defense strategy in response to pathogens invasion. The significant differences were found on the superoxide dismutase (SOD), peroxidase (POD), catalase (CAT), and ascorbate peroxidase (APX) enzyme activities, both in leaves and roots of tomato seedlings planted in inoculated bacteria (Peat + RS and VRS + RS) compared to uninoculated bacteria (Peat and VRS) (Figure 6). After inoculating with RS, the activities of these four enzymes mentioned above in tomato roots in VRS + RS were 0.71-, 0.15-, 0.50-, and 0.82-fold, respectively, higher than in Peat + RS treatment (Figure 5). Nevertheless, POD activity in VRS + RS-treated tomato leaves were markedly decreased in comparison to the Peat + RS, and the CAT and APX activities of the leaves showed no noticeable differences (Figure 6). 

**Figure 7:**
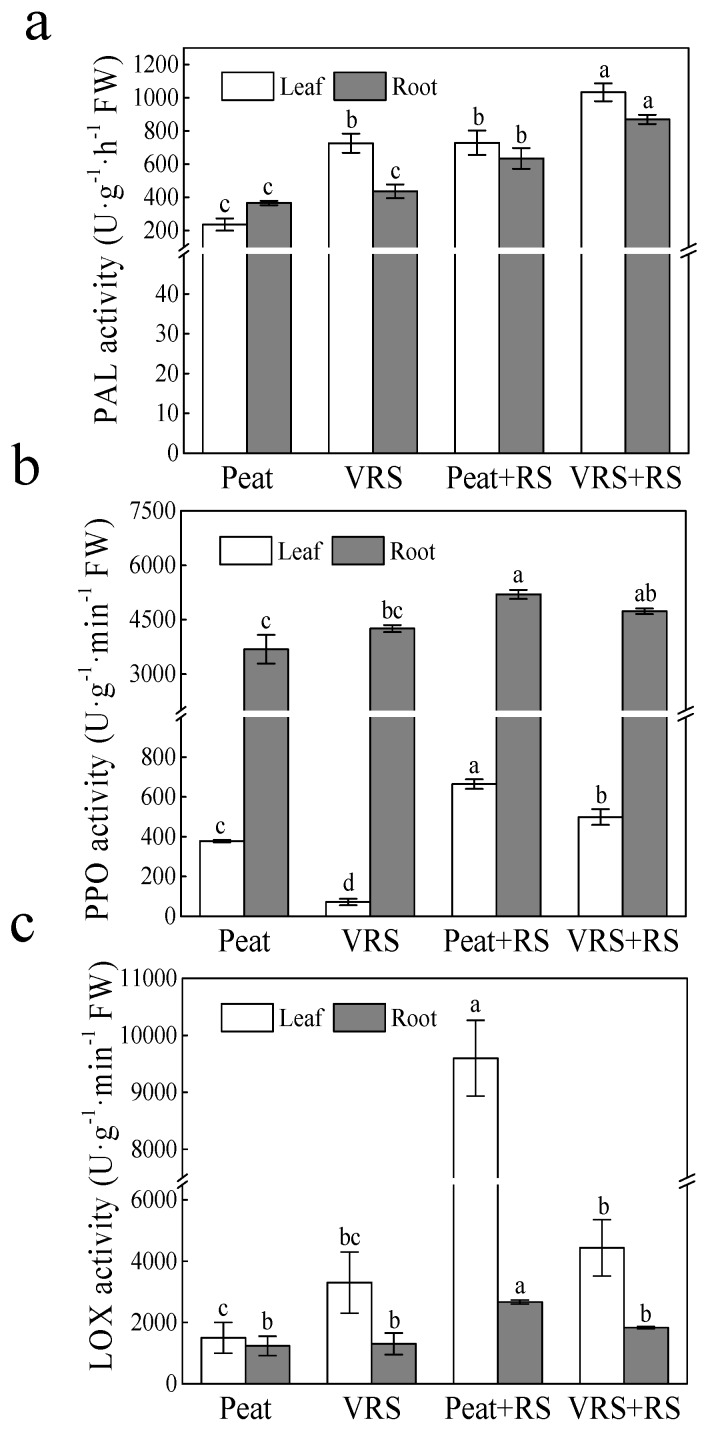
Effects of different growth media on (**a**) phenylalanine ammonia lyase (PAL), (**b**) polyphenol oxidase (PPO) and (**c**) lipoxygenase (LOX) enzyme activities in leaves and roots of tomato seedlings after pathogenic bacteria treatments. Peat = peat and vermiculite in a 2:1 ratio (v/v), VRS = vinegar residue substrate, vinegar residue/peat/vermiculite = 3:2:1 (v/v/v). Peat + RS (*Ralstonia solanacearum*) = peat inoculated with pathogen, VRS + RS = VRS poured with pathogen suspensions. Different letters indicate the significant differences among the treatments at the level of *p* < 0.05, according to Duncan’s multiple range tests. Three independent biological replicates were carried out with similar results.

As shown in Figure 7, in comparison without RS root-irrigation, the PAL, PPO, and LOX enzyme activities, both in tomato leaves and roots, showed a prominent rise or an uptrend after RS root-irrigation. In contrast to Peat + RS, the PAL activity in VRS + RS-treated plants increased by 29.5% and 27.0% in leaves and roots, respectively (Figure 7a). Analogously, the activities of PPO and LOX in leaves of VRS + RS-treated plants reduced by 24.9%, and 53.8% than the plants existed in Peat + RS, respectively, and the PPO and LOX activities in roots also declined by 9.0% and 31.3%, respectively (Figure 7b,c). 

### 2.8. The Expression Patterns of Defense Marker Genes 

**Figure 8:**
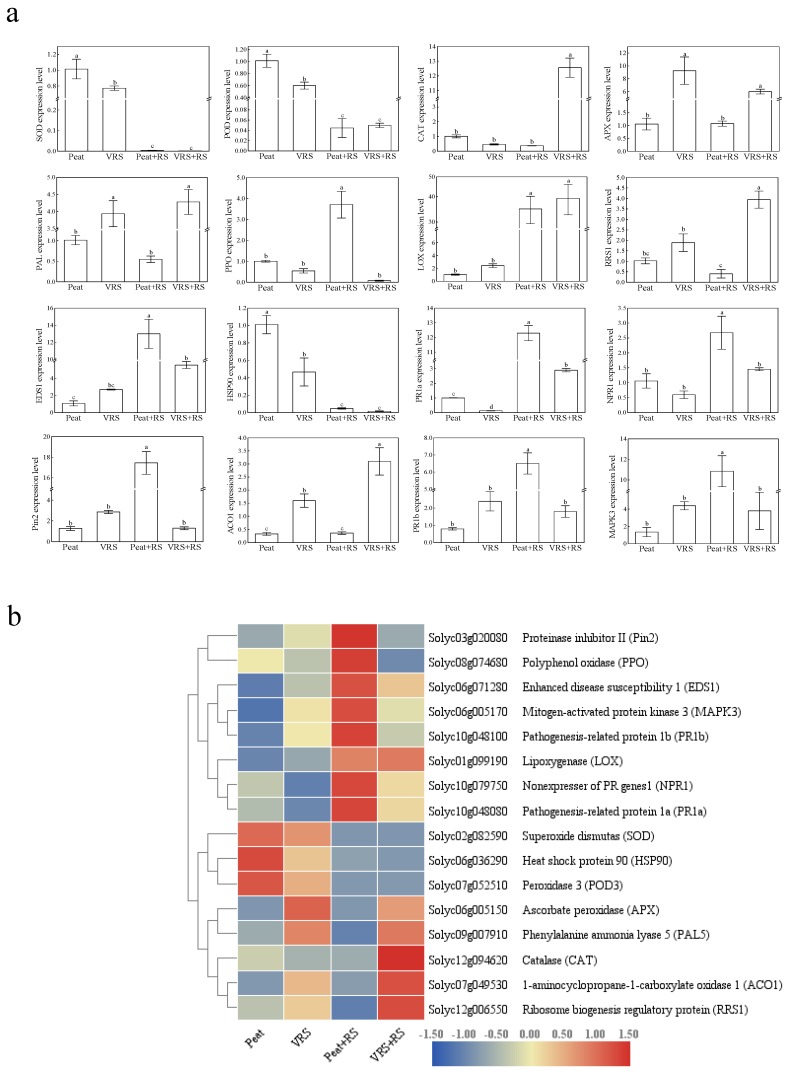
Real-time quantitative polymerase chain reaction (qRT-PCR) analysis of defense-related gene expression in leaves of tomato seedlings after challenging with pathogen in different compost treatments (**a**). The gene expression values of stress-related genes were indicated by using a heat map and were used in a hierarchical cluster analysis (**b**). Red color represents higher relative expression, and blue color represents lower relative expression when compared with the control samples. Peat = peat and vermiculite in a 2:1 ratio (v/v), VRS = vinegar residue substrate, vinegar residue/peat/vermiculite = 3:2:1 (v/v/v). Peat + RS (*Ralstonia solanacearum*) = peat inoculated with pathogen, VRS + RS = VRS poured with pathogen suspensions. Different letters indicate the significant differences among the treatments at the level of *p* < 0.05, according to Duncan’s multiple range tests. Three independent biological replicates were carried out with similar results.

For further insights into the regulatory mechanism on how VRS modified plants to control bacterial wilt, we quantified different defensive marker genes expression. The transcription levels of *APX*, *PAL*, *LOX*, *ACO1*, *EDS1*, *PR1B*, *MAPK3*, *PIN2*, and *RRS1* in VRS-planted tomato leaves were up-regulated, importantly, the expression level of *APX*, *PAL*, and *ACO1* genes noticeably increased by 7.73-, 2.90-, and 3.82-fold, respectively, higher than Peat-treated plants (Figure 8). The expression patterns of *APX*, *LOX*, *EDS1*, *PR1a*, *NPR1*, and *ACO1* further up-regulated in Peat + RS and VRS + RS treatments in comparison to Peat and VRS. However, the gene expression of *SOD*, *POD*, and *HSP90* in Peat + RS-treated seedlings down-regulated by 99.6%, 95.5%, and 95.2%, respectively, and down-regulated by 99.8%, 91.7%, and 96.5%% in VRS + RS-treated seedlings, respectively (Figure 8). Compared with Peat + RS, the tomato planted in VRS infected with RS could irritate the expression levels of these genes (*CAT*, *POD*, *APX*, *PAL*, *LOX*, *RRS1*, and *ACO1*), and remaining gene expression levels suffered from down-regulating to varying degrees (Figure 8). Nevertheless, the *PPO* and *PIN2* genes emerged with notably lower transcription levels, with 97.9% and 92.5% reduction, respectively (Figure 8). 

## 3. Discussion

Soil-borne diseases have an adverse effect on plant yield and quality [18,19], and compost and an organic matrix provide an effective strategy for controlling disease occurrence [20,21,22]. The VRS tested in this research significantly suppressed tomato bacterial wilt (Figure 2). The present study results are in accordance with those of Du et al. [14], who reported that VRS showed superior suppression of cucumber fusarium wilt. Besides, tomato growth parameters and root morphological indices were all lower in Peat + RS and VRS + RS treatments (Table 1 and Table 2). However, tomato seedlings grew better in VRS than in Peat whether or not RS inoculation occurred. These findings indicated that VRS not only suppressed the bacterial wilt infestation but also prompted plant growth. This is consistent with the results of compost’s function in controlling tomato bacterial wilt proposed by Schönfeld et al. [23].

The culture conditions of traditional microbial culture methods are confined. Therefore, the conventional microbial culture method is only suitable as an auxiliary means in the study of microbial community structure [24]. It is known that the application of organic matter to soil promotes microbial communities and biological activity in the soil [25,26]. Our study showed that the VRS application significantly boosted the bacteria and actinomycetes populations compared to the control, but markedly decreased the fungal population (Figure 3). This result was in accord with the findings of Brussaard et al. [27], who stated that fungi could cause more damage, and some bacteria carry the potential to suppress pathogens. Furthermore, VRS prominently reduced the population of RS relative to conventional substrates. These data demonstrated that VRS modified the composition of microbial communities, markedly reduced RS population, showed lower disease incidence, and advanced plant health, which coincided with the effect of compost on bacterial wilt suppression of potato [28] and tobacco [29]. Microorganisms possibly cause the impact of disease suppression in competing with pathogens for nutrients and the ecological niche. 

Soil enzymes perform an integral role in facilitating biochemical transformations containing decomposition of organic residues and the cycling of nutrients in the soil [25]. In this study, the application of vinegar residue amendments in soil notably increased the soil enzyme activities (Table 3), indicating a positive relationship with soil fertility, as well as showing that VRS can improve the rhizosphere environment. Among these enzymes, the activities of FDA hydrolysis and β-glucosidase are seen as potential predictive arguments for general inhibition, because they can manifest nutritional competition [14]. VRS is affluent in micronutrients and helpful as a disease-inhibiting growth-promoting medium because it maintains a higher degree of microbial community and microbial activity. 

A series of experimental shreds of evidence certified that organic compost could play critical roles as an antioxidant and growth stimulator, particularly in stress environments [30], and compost is regarded as a promising natural material for agricultural use. In the present experiment, we found that pathogen attack significantly induced higher levels of ROS in terms of O_2_^•−^ and H_2_O_2_ production (Figure 4), which resulted in higher EL and MDA contents (Figure 5); similar results were obtained in other studies [31,32]. The oxidative stress induced by biotic stress was alleviated by VRS (Figure 4 and Figure 5). Disorders of ROS metabolism in plants could be promotes excess ROS production. Notably, the key ROS-scavenging enzymes SOD, APX, POD, and CAT in plants participated in the cellular defense systems. Lower antioxidant enzyme activities could be responsible for the accumulation of ROS in RS-treated tomato seedlings, and higher antioxidant enzyme activities could somehow impart plant resistance to pathogen infection. In this present research, the antioxidant enzymes activities in plants transplanted in growing media challenged with RS (Peat + RS and VRS + RS) were higher than those in media without RS (Peat and VRS) (Figure 6). These research results are consistent with earlier reports that the activity of antioxidant enzymes is increasing and remains at a high level in stressed plants to eliminate reactive oxygen damage.

As key elements in the defense mechanisms, the cell metabolism is significantly induced, primarily in the defense-related enzymes, including PAL, PPO, and LOX. PAL contributed to the biosynthesis of phenolics, phytoalexins, and lignins as well as other substances associated with the resistance of localized diseases in plants [33]. Shi et al. [30] proposed that, in cucumber seedlings, PAL possessed relatively high resistance to disease shown by VRS through the use of the secondary metabolic pathways. This finding is analogous to our results, wherein PAL activity in response to RS inoculation was enhanced and was better in VRS than those in Peat (Figure 7a). Likewise, Umesha [34] also addressed the role of PAL in the provision of tomato resistance to bacterial cancer diseases. PPO is involved in host plant cell lignification and is considered as a key enzyme linked to the protective reaction against pathogen infections [35]. LOX catalyzes the production of hydroperoxides and lipid-containing free radicals produced by lipid peroxidation, which is involved in signal transduction and plays a vital role in resisting environmental stress [36]. According to the results depicted above (Figure 7b,c), the activities of PPO and LOX increased after inoculation with RS. Furthermore, the PPO and LOX activities in the tomato seedlings leaves and roots cultured in VRS + RS treatment were lower than in Peat + RS, but the PPO activity in root was not significant. These suggested that PPO and LOX are usually induced to a different degree when pathogens enter the plant. The decrease of PPO and LOX activities may be associated with the reduction of membrane lipid peroxidation, indicating that the defense-related enzymes play a vital role in pathogen shock. 

Furthermore, *RRS1* confers resistance to RS [37]. In leaves, the *RRS1* expression level in the cultured in VRS + RS was substantially higher than cultured in Peat + RS (Figure 8) Besides, the transcription level of *RRS1* in Peat upon RS inoculation was lowest in these four treatments. These illustrated that VRS could induce *RRS1* expression to improve the resistance to bacterial wilt when RS infects the plants. However, Peat + RS-treated tomato seedlings are injured so severely that the plants cannot adequately express *RRS1* to fight against disease attack. These results are in accord with Tasset et al. [38] who found that *RRS1* can activate the plant immune response upon RS infection. 

Adequate stimuli or signals are required for the activation of defense-related genes. It has been shown that phytohormones salicylic acid (SA), jasmonic acid (JA), and ethylene (ET) play a critical role in signaling networks that regulate plant-induced resistance [39]. In this experiment, the defense marker genes *PR1a*, *NPR1*, *PIN2*, *ACO1*, and *PR1b*, which, respectively, encode the enzymes to the SA, JA, and ET signaling pathways, were differentially expressed in different manner after the plants were subjected to pathogen inoculation, implying that systemic-acquired resistance possesses cross-talk between SA-, JA-, and ET-dependent signaling pathways. After the RS infection, transcription expression levels of *PR1a*, *NPR1*, *PIN2,* and *PR1b* in Peat + RS were prominently upregulated than that in VRS + RS; otherwise, no responses consisted of other treatments upon *NPR1*, *PIN2,* and *PR1b*. These data make clear that pathogens simultaneously activate multi-term signaling pathways. However, with the VRS application, none of *NPR1*, *PIN2,* and *PR1b* responded to RS infection, which implied that VRS inhibition of RS involved an intricate mechanism that required further research. In addition, heat mapping identified analogous trends in *EDS1* and *MAPK3* expressions that are necessary to withstand RS injuries [40]. Analogously, the transcriptional level of *HSP90,* which has been reported to have a negative regulatory effect on bacterial wilt in tobacco [41], is similar to the expression level of antioxidants. 

## 4. Materials and Methods

### 4.1. Plant, Bacterial Strains, and Growth Condition

Tomato (*Solanum lycopersicum* L) “hezuo 903”, a susceptible cultivar to bacterial wilt, was used as the plant material. Peat, vermiculite, and vinegar residue compost were used as nursery substrates and provided by Peilei Zhenjiang Company (Nanjing, Jiangsu, China). Nutrient agar medium (YGPA, glucose 10.0 g, peptone 5.0 g, H_2_O 1 L, yeast extract 5.0 g, pH 7.0–7.2) was used to grow *R. solanacearum* HB511 strain and maintained in YGPA broth supplemented with 40% glycerol at −80 °C as storing stocks. For routine use, the glycerol bacteria was streaked on the plate, after culturing for 2–3 days at 28 °C on YGPA plates. Then the bacteria were picked to transfer to YGPA liquid culture (without agar) for 18–24 h of shaking (200 rpm·min^−1^) in incubator shaker at 28 °C, and the bacterial suspensions with the concentration of OD_600_ = 0.8 (about 1.0 × 10^8^ CFU·mL^−1^) were collected for further use.

### 4.2. Pot Experiment and Inoculation with RS

A pot experiment was carried out in the greenhouse where temperature was maintained at 25–30 °C in the daytime and 15–18 °C in darkness, with the relative humidity between 60%–70%. Tomato seeds were surface-sterilized for 5 min with 3% NaOCl (sodium hypochloride) solution and washed with distilled water several times and placed in a humid environment to germinate at 28 °C for 48 h. The germinated seeds were subsequently sown in a tray containing a nursery substrate. When entirely second true leaves were developed they were transplanted into the nutrient pot (110 × 90 cm). We designed the pot experiment treatments as follows: (i) Peat and vermiculite (2:1) as the treatment of control (Peat); (ii) vinegar residue compost, peat, and vermiculite mixtures at a proportion of 3:2:1 as the treatment of vinegar residue substrate (VRS); (iii) peat inoculated with pathogenic bacteria denoted as Peat + RS; (iv) VRS inoculated with pathogens indicated as VRS + RS. 

We compared the virulence of RS among the different substrates in tomato plants employing a root-injured irrigation inoculation [42]. Briefly, for root wounding irrigation inoculation assay, the wounded-root tomato plants were treated by pouring 20 mL bacterial suspensions (10^8^ CFU·mL^−1^) of RS, and, for control, sterilized water was used into the substrate per pot. After inoculation, plants were transferred in a growth chamber where they were maintained at a temperature of 28 °C, a photoperiod of 12:12 h, and a humidity of 90% to 95%. 

### 4.3. Monitoring and Evaluation of Disease Symptoms

After infection, seedlings were observed regularly, and symptoms of the diseases were recorded on an index scale of 0 to 4 as described by Milling et al. [43], in which 0 meant no wilting, 1 indicated 1%–25% of leaves wilted, 2 indicated 26%–50% of leaves wilted, 3 indicated 51%–75% of leaves wilted, and 4 indicated 76%–100% of leaves wilted or dead. Disease severity index = {(the number of diseased plants in this index × disease index)/ (total number of plants investigated × highest disease index)} × 100%. At least 16 plants were used to assay the virulence for each treatment and repeated thrice. Tomato seedlings were sampled ten days after inoculation, and stored at −80 °C for subsequent chemical analysis.

### 4.4. Analysis of Basic Physical and Chemical Properties of the Matrix

We estimated the pH and EC of the substrates using the methodology of Hidalgo, et al. [44]. The bulk density and porosity of tested materials were determined by the method of Shi et al. [45]. The air-dried substrates were dehydrated by the H_2_SO_4_-H_2_O_2_ digestion method, and the Kjeldahl method [46] was used to estimate the total nitrogen. The content of P, K, Ca, Na, and Mg nutrient elements were determined by inductively-coupled plasma emission spectrometer (Perkin Elmer Optima 2100DV) [47]. The main physicochemical properties of materials are shown in Table 4.

### 4.5. Evaluation of the Plants’ Growth-Promoting Properties In Vivo

After ten days of inoculation, we determined the plant growth indices like plant height, stem diameter, and leaf areas following the standard methods. To determine the fresh and dry mass of plants, the shoot and root systems of uprooted plants were separated, and the samples were heat-treated at 105 °C for 15 min, the temperature was then adjusted to 75 °C until the weight was constant. The root morphology index and root vigor of tomato seedlings were detected with the combined methods of root scanning (Expression 12000 XL) and Triphenyl tetrazolium chloride (TTC) [48].

### 4.6. Analysis of Microbial Community Populations and Soil Microbial Activities

The dilution plate method was used to calculate the culturable bacteria, fungi, actinomycetes, and RS. The colony of bacteria was measured by coating 100 μL of suspensions on beef paste peptone solid medium. The quantity of the fungus colony-forming units was formed on Martin’s agar medium with 1.25 g L^−1^ streptomycin and 33 mg L^−1^ Rose Bengal. CFUs populations of actinomycetes were counted on the Gause NO.1 agar medium [49]. The populations of RS were estimated employing a modified semi-selective medium as described by French [50]. The inoculated plate was cultured in a biochemical incubator, and colonies were counted and expressed as CFU per gram of growth substrate.

Invertase activity in the soil was determined with a spectrophotometer using the method of 3,5-di-nitrosalicylic acid described by Hou et al. [51]. The activity of urease was denoted as mg NH_4_^+^ -N released per gram dried soil during 24 h incubation at 30 °C [52]. Proteinase activity was measured spectrophotometrically using ninhydrin at 500 nm [53]. Catalase activity was performed by titration method with 0.1 M KMnO_4_ [54]. Neutral phosphatase activity was assayed by colorimetric analysis using the sodium phenyl phosphate method [54]. The activity of β-glucosidase and FDA hydrolysis was acquired by the protocol developed by Hayano [55] and Adam and Duncan [56].

### 4.7. Determination of Electrolyte Leakage and Lipid Peroxidation

The relative electrolyte leakage (EL) of leaves was estimated according to Jahan et al. [57]. Briefly, 0.5 g fresh leaves were taken and thoroughly washed with deionized water, then placed into 20 mL tubes containing deionized water. The samples were kept at room temperature for 2 h and shaken several times. Primarily, initial electrical conductivity (EC_1_) of these solutions were determined by a portable conductivity meter. The leaf samples were boiled for 10 min and kept in room temperature until cool to calculate the final electrical conductivity (EC_2_). Simultaneously, we estimated the conductivity of deionized water (EC_0_). To determine the electrolyte leakage, the following formula was used: EL = (EC_1_ − EC_0_)/(EC_2_ − EC_0_) × 100%.

Lipid peroxidation was monitored by estimating the malondialdehyde content (MDA) in leaves (0.2 g), according to KoÇ [58]. 

### 4.8. Analysis of ROS Accumulation in Pathogen Inoculated Leaves

According to Jambunathan [59] was used to detect the H_2_O_2_ and O_2_^•−^ through leaf staining method. For the H_2_O_2_ staining, tomato leaves were vacuum infiltrated with 1 mg mL^−1^ DAB in 50 mM Tris-HCl (pH 3.8) and incubated at room temperature under illumination for 24 h. For the O_2_^•−^ staining, leaves were vacuum infiltrated with 0.1 mg·mL^−1^ NBT in 25 mM HEPES buffer (pH 7.8) and hatched at 25 °C in the dark for 6 h. For both, the chlorophyll was removed by boiling the stained leaf samples for 30 min and placed in absolute ethanol overnight. Representative photographs of the staining leaf of DAB and NBT were taken with a light microscope. The H_2_O_2_ content was estimated according to Wu et al. [60]_._ The technique described by Elstner and Heupel [61] was applied to analyze the production of O_2_^•−^. 

### 4.9. Assay of Enzyme Activities

The frozen samples of tomato both leaves and roots (0.2 g) were ground using a precooled mortar in 1.6 mL of cold PBS (0.05 M, pH 7.8) and followed by centrifugation at 12,000× *g* for 20 min at 4 °C, which were used to assay antioxidant enzyme activities. Superoxide dismutase (SOD) activity was estimated with the method published by Giannopolitis and Ries [62]. Peroxidase (POD) activity was evaluated as described by Nickel and Cunningham [63]. Catalase (CAT) activity was measured with the protocol developed by [64]. The method of Nakano and Asada [65] was used to examine ascorbate peroxidase (APX) activity. The activity of Phenylalanine ammonia-Lyase (PAL) was assayed, according to Assis et al. [66]. The determination of polyphenol oxidase (PPO) activity depended on the technique of González et al. [67]. Lipoxygenase (LOX) activity was measured with the lipoxygenase assay kit (Solarbio Life Science, Beijing, China).

### 4.10. Analysis of Defense-Related Genes Expression

Total RNA was extracted (0.1 g) using the TotalRNA Kit (Tiangen, Beijing, China) following the manufacturer’s instructions. A total of 1 μg of RNA was reverse transcribed to make cDNA with the help of the superscript first-strand synthesis system for qRT-PCR (Takara, Tokyo, Japan). The primers were designed based on the sequences required from searching for the NCBI database and Sol Genomics Network (solgenomics.net). Gene-specific primers were used to quantify the genes (Table 5). The qRT-PCR assays were performed using a StepOnePlus™ Real-Time PCR System (Applied Biosystems, Foster city, CA, USA) with a ChamQ Universal SYBR qPCR Master Mix (Vazyme Biotech Co. Ltd., Nanjing, China). The reactions needed three biological replicates and the thermocycler conditions presented as follows: A temperature of 95 °C for 5 min for denaturation followed by 40 cycles at 95 °C for 15 s, annealing at 60 °C for 1 min, and a final extension of 15 s at 95 °C. Relative gene expression was calculated using the formula of 2^−ΔΔCt^ [68], where the relative degree of expression against the actin was normalized and compared to the control.

### 4.11. Statistical Analysis

Independent three biological replications were used for each treatment parameter determination. Statistical analysis was carried out with the SPSS 18 statistical software package. The rhizosphere colonization data were converted to log 10 values, and data were analyzed with Duncan multiple range test at *p* < 0.05. TBtools statistics software was used to construct a heatmap for transcript expression.

## 5. Conclusions

The core findings of the present research enriched our understanding that VRS is a potential organic substrate for controlling bacterial wilt in tomato seedlings through the suppression of disease incidence, and changing the activity both of the soil enzymes and microbials community. Besides, VRS prominently enhanced the activities of invertase, urease, proteinase, and β-glucosidase. VRS significantly inhibited disease-induced oxidative damage via excess ROS (H_2_O_2_, O_2_^•−^) scavenging, and increased antioxidant defense capacity. Antioxidant encoding enzymes as well as defense-related genes expression were also upregulated by VRS treated seedlings. All of these together concluded that VRS could effectively be prompted the tomato seedling growth and enhanced resistance to bacterial wilt. For insights into the precise mechanism of VRS’s function against bacterial wilt, a molecular research approach could be applied.

## Figures and Tables

**Table 1:** Effects of different compost treatments on growth index of tomato seedlings exposed for 10 days to *R. solanacearum* HB511.

Treatment	Plant Height (cm)	Stem Diameter (mm)	Leaf Area (cm^2^)	Shoot Fresh Weight (g)	Root Fresh Weight (g)	Shoot Dry Weight (g)	Root Dry Weight (g)
Peat	15 ± 0.24 ^b^	3.81 ± 0.03 ^b^	20.84 ± 0.85 ^b^	4.23 ± 0.32 ^b^	0.86 ± 0.03 ^b^	0.36 ± 0.03 ^b^	0.07 ± 0.003 ^b^
VRS	16.2 ± 0.12 ^a^	3.95 ± 0.01 ^a^	25.17 ± 0.21 ^a^	5.20 ± 0.36 ^a^	1.05 ± 0.08 ^a^	0.45 ± 0.03 ^a^	0.10 ± 0.003 ^a^
Peat + RS	9.84 ± 0.26 ^d^	3.41 ± 0.02 ^d^	10.09 ± 0.63 ^d^	2.06 ± 0.02 ^d^	0.59 ± 0.02 ^d^	0.21 ± 0.006 ^c^	0.04 ± 0.003 ^d^
VRS + RS	11.31 ± 0.50 ^c^	3.59 ± 0.03 ^c^	13.32 ± 0.61 ^c^	3.06 ± 0.09 ^c^	0.74 ± 0.03 ^c^	0.26 ± 0.006 ^c^	0.06 ± 0.003 ^c^

Note: Peat = peat and vermiculite in a 2:1 ratio (v/v), VRS = vinegar residue substrate, vinegar residue/peat/vermiculite = 3:2:1 (v/v/v). Peat + RS (*Ralstonia solanacearum*) = peat inoculated with pathogen, VRS + RS = VRS poured with pathogen suspensions. Different letters indicate the significant differences among the treatments in the same column at *p* < 0.05, according to Duncan’s multiple range tests. Three independent biological replicates were carried out with similar results.

**Table 2:** Effects of different compost treatments on root morphology of tomato seedlings exposed for 10 days to *R. solanacearum* HB511.

Treatment	Total Root Length (cm)	Root Surface Area (cm^2^)	Root Volume (cm^3^)	Mean Diameter (mm)	Tips of Number	Root Vigor (mg·g^−1^·h^−1^)
Peat	461.6 ± 8.7 ^b^	54.42 ± 4.17 ^b^	0.52 ± 0.08 ^ab^	0.38 ± 0.03 ^c^	1164 ± 54 ^b^	74.51 ± 2.72 ^a^
VRS	611.9 ± 22.6 ^a^	69.29 ± 1.22 ^a^	0.63 ± 0.04 ^a^	0.36 ± 0.02 ^bc^	1370 ± 7 ^a^	84.69 ± 3.68 ^a^
Peat + RS	258.6 ± 20.2 ^c^	37.46 ± 0.28 ^c^	0.44 ± 0.03 ^b^	0.47 ± 0.04 ^a^	597 ± 33 ^c^	27.23 ± 1.89 ^c^
VRS + RS	274.0 ± 7.69 ^c^	37.85 ± 0.37 ^c^	0.42 ± 0.01 ^b^	0.44 ± 0.01 ^ab^	637 ± 84 ^c^	62.13 ± 3.15 ^b^

Note: Peat = peat and vermiculite in a 2:1 ratio (v/v), VRS = vinegar residue substrate, vinegar residue/peat/vermiculite = 3:2:1 (v/v/v). Peat + RS (*Ralstonia solanacearum*) = peat inoculated with pathogen, VRS + RS = VRS poured with pathogen suspensions. Different letters indicate the significant differences among the treatments in the same column at *p* < 0.05, according to Duncan’s multiple range tests. Three independent biological replicates were carried out with similar results.

**Table 3:** The effect of RS on the enzyme activities of different growth media on day 10 after RS infection.

Treatment	Invertase (mg glucose g^−1^ h^−1^)	Urease (mg NH_4_^+^ -N g^−1^ h^−1^)	Proteinase (mg glycine kg^−1^ h^−1^)	Catalase (mL (0.1 M KMnO_4_) g^−1^ h^−1^)	Phosphatase (mg phenol g^−1^ h^−1^)	β-glucosidase (μg hydrolyzed p-nitrophenol g^−1^ h^−1^)	FDA hydrolysis (μg FDA g^−1^ h^−1^)
Peat	11.20 ± 2.08 ^bc^	0.91 ± 0.03 ^c^	2.54 ± 0.22 ^c^	2.96 ± 0.008 ^b^	0.49 ± 0.01 ^a^	706.8 ± 19.59 ^c^	925.6 ± 8.49 ^c^
VRS	39.99 ± 4.42 ^a^	1.95 ± 0.09 ^a^	10.79 ± 0.35 ^a^	3.15 ± 0.006 ^a^	0.57 ± 0.006 ^a^	1532.3 ± 15.69 ^a^	1387.1 ± 22.86 ^a^
Peat + RS	4.82 ± 0.27 ^c^	0.10 ± 0.03 ^c^	0.72 ± 0.06 ^d^	2.86 ± 0.057 ^b^	0.38 ± 0.04 ^b^	654.2 ± 54.09 ^c^	911.7 ± 12.14 ^c^
VRS + RS	13.89 ± 1.68 ^b^	1.25 ± 0.06 ^b^	6.98 ± 0.06 ^b^	3.15 ± 0.006 ^a^	0.52 ± 0.005 ^a^	1379.2 ± 12.66 ^b^	955.8 ± 13.69 ^b^

Note: Peat = peat and vermiculite in a 2:1 ratio (v/v), VRS = vinegar residue substrate, vinegar residue/peat/vermiculite = 3:2:1 (v/v/v). Peat + RS (*Ralstonia solanacearum*) = peat inoculated with pathogen, VRS + RS = VRS poured with pathogen suspensions. Different letters indicate the significant differences among the treatments in the same column at *p* < 0.05, according to Duncan’s multiple range tests. Three independent biological replicates were carried out with similar results.

**Table 4:** Physical and chemical characteristics of compound substrates used in this study.

	Peat	VRS
pH	5.16 ± 0.04	5.88 ± 0.16
EC (ms·cm^−1^)	0.77 ± 0.12	1.62 ± 0.30
Total porosity (%)	80.5 ± 0.51	80.1 ± 0.44
Aeration porosity (%)	1.72 ± 0.57	9.28 ± 0.08
Water-holding porosity (%)	78.8 ± 0.58	70.9 ± 0.46
Aeration porosity/Water-holding porosity	2.18 ± 0.74	13.1 ± 0.16
Bulk density (g·cm^-3^)	18.7 ± 0.05	12.2 ± 0.26
Total N (mg·g^−1^)	13.0 ± 0.70	18.4 ± 1.11
Total P (mg·g^−1^)	1.00 ± 0.05	4.71 ± 0.14
Total K (mg·g^−1^)	1.12 ± 0.20	4.30 ± 1.50
Na (mg·g^−1^)	0.46 ± 0.05	1.08 ± 0.34
Ca (mg·g^−1^)	3.31 ± 0.77	7.93 ± 2.01
Mg (mg·g^−1^)	0.42 ± 0.15	2.26 ± 0.76

Note: Peat = peat and vermiculite in a 2:1 ratio (v/v), VRS = vinegar residue substrate, vinegar residue/peat/vermiculite = 3:2:1 (v/v/v). Three independent replicates were carried out with similar results.

**Table 5:** Primers used for qRT-PCR assays.

Gene Full Name	Gene Acronym	Accession Numbers	Forward Primer	Reverse Primer
*Superoxide dismutase*	*SOD*	Solyc02g082590	5′-ATAGGAAGCCATACGATA-3′	5′-ATCACCGCATATTGTAAT-3′
*Peroxidase 3*	*POD3*	Solyc07g052510	5′-CTGGTAGAAGAGATGGAA-3′	5′-CGAAGGATTGTTGTAGTC-3′
*Catalase*	*CAT*	Solyc12g094620	5′-ATTCCTTCTTGTGTCTTG-3′	5′-TGTTGATGTATCTGTCTTG-3′
*Ascorbate peroxidase*	*APX*	Solyc06g005150	5′-CCTATGATGTGTGTTCCA-3′	5′-AAGAGTCTGAGAGCAATG-3′
*Phenylalanine ammonia lyase 5*	*PAL5*	Solyc09g007910	5′-CGGTGAGGAGATTGATAA-3′	5′-TTAGCAGATTGGAATAGGA-3′
*Polyphenol oxidase*	*PPO*	Solyc08g074680	5′-TACTACTACAACGCTCAA-3′	5′-AACCAAGAAGAACATTCC-3′
*Lipoxygenase*	*LOX*	Solyc01g099190	5′-TTGGCTTATACTCTTACG-3′	5′-GAATACCTTGTCTGGATT-3′
*1-aminocyclopropane−1-carboxylate oxidase 1*	*ACO1*	Solyc07g049530	5′-TTGACGAAGAATACAGAGA-3′	5′-ATGGTGGATAGTTGCTAA-3′
*Mitogen-activated protein kinase 3*	*MAPK3*	Solyc06g005170	5′-ATGGTTGATGCTAATATGG-3′	5′-AGGAGGTTGATACTTGTT-3′
*A key regulator of the SA-mediated systemic-acquired resistance pathway*	*NPR1*	Solyc10g079750	5′- GCGATATTCCAACCTATA-3′	5′-TAGATTCAAATACACCATTC-3′
*Enhanced disease susceptibility 1*	*EDS1*	Solyc06g071280	5′-AATGATGCTTGCTCCTCTT-3′	5′-GCCTCGTGCTGATAATACT-3′
*Ribosome biogenesis regulatory protein homolog*	*RRS1*	Solyc12g006550	5′-TTGGTGAAGGAGTGTCTA-3′	5′-TCTGTTGAAGGTAAGTTGAA-3′
*Heat shock protein 90*	*HSP90*	Solyc06g036290	5′-TGTTGTTGACTCTGATGATT-3′	5′-GTTCTTCCTAATGACCTTGA-3′
*Pathogenesis-related protein 1a*	*PR1a*	Solyc10g048080	5′-GCTCATCCAAATAGTATCC-3′	5′-GGTCTAACTCCCACATTA-3′
*Pathogenesis-related protein 1b*	*PR1b*	Solyc10g048100	5′-ATTCTCATGGTCAGTATT-3′	5′-GGTAATAGTATTGTTTCTCA-3′
*Proteinase inhibitor II*	*Pin2*	Solyc03g020080	5′-TGATGCCAAGGCTTGTACTAGAGA-3′	5′-AGCGGACTTCCTTCTGAACGT-3′
